# The Primary Management Strategies for ST-Elevation Myocardial Infarction Patients in Saudi Arabia: A Sub-Study of the Saudi Acute Myocardial Infarction Registry

**DOI:** 10.7759/cureus.11783

**Published:** 2020-11-30

**Authors:** Abdulhalim J Kinsara, Ayman Alsaleh, Ziad A Taher, Mostafa Alshamiri, Fayez Elshaer

**Affiliations:** 1 Cardiology, Ministry of National Guard - Health Affairs, King Saud Bin Abdulaziz University for Health Sciences, COM-WR, King Abdullah International Medical Research Center, Jeddah, SAU; 2 Cardiology, Department of Cardiac Sciences, King Fahad Cardiac Center, College of Medicine, King Saud University, Riyadh, SAU; 3 Internal Medicine, Department of Medicine, Ministry of National Guard - Health Affairs, Jeddah, SAU; 4 Internal Medicine, Department of Medicine, King Abdullah International Medical Research Center, Jeddah, SAU

**Keywords:** st-elevation myocardial infarction (stemi), primary percutaneous coronary intervention, thrombolytic therapy, coronary revascularization

## Abstract

Background and objective

Not all patients with ST-elevation myocardial infarction (STEMI) in Saudi Arabia are managed with a primary percutaneous coronary intervention (PPCI). We analyzed the management strategies for STEMI patients in the Saudi Acute Myocardial Infarction Registry (STARS). The strategies include PPCI, revascularization with thrombolytic therapy, and conservative management. This study involved a sub-study of the STARS.

Methods

STEMI patients were categorized into three groups. Group 1 was managed with PPCI, group 2 with revascularization with thrombolytic therapy, and group 3 with conservative approaches. The data were collected at presentation, at one month, and at one year after discharge.

Results

The sample consisted of 1,471 patients. The mean age of the participants was 54 ±12 years; 51% were Saudi citizens, and the majority (89%) were male. Their background revealed a high coronary risk profile, with 48% diagnosed with diabetes mellitus (DM) and 44% with hypertension (HTN); 54% were active or ex-smokers, 30% had a high lipid profile, and 74% were overweight. PPCI was performed in 42%, and 29% were managed with revascularization using thrombolytic therapy. A conservative approach was followed in 29% of the patients. Patients who had a stroke were treated conservatively due to the risk of bleeding. The patients in group 1 were mostly hypertensive with recurrent angina and a history of prior revascularization, with PPCI or coronary artery bypass grafting (CABG). The crude all-cause mortality at one year was 11%; it was 7% at one month for group 1, 8% for group 2, and 9% for group 3, which was not statistically significant.

Conclusions

Controlling the risk factors and improving access to PPCI in hospitals are fundamental in the management of STEMI patients. PPCI is still underused. Guideline-directed medical therapy (GDMT) is a reasonable approach if PPCI is not available.

## Introduction

ST-elevation myocardial infarction (STEMI) is a major challenge for health authorities [1]. The option of primary percutaneous coronary intervention (PPCI) is not available in all hospitals or at all times. The ideal time for the management is limited and affected by the level of the patient’s knowledge about the symptoms, the availability of transport services to move the patients to the right centers, and the transmission of ECG en route to the PPCI center. Providing an on-call service 24 hours, seven days a week is costly and demanding for the intervention cardiologist [2]. Many small hospitals have elected to use only thrombolytic therapy (conservative approach) or “drip and ship” or, rarely, rescue PPCI if the thrombolysis fails based on the hemodynamic status of the STEMI patient. The choice of strategy is occasionally limited due to the comorbid conditions associated with a higher risk of bleeding or a limitation for long-term antiplatelet medication, for example, patients with a brain tumor or recent major surgery [3]. We prospectively evaluated the outcome of patients who presented with STEMI in hospitals with or without a cardiac catheterization lab and compared the outcomes of the management strategies for STEMI patients in Saudi Arabia.

## Materials and methods

From May 2015 to January 2017, 2,233 patients with acute coronary syndrome were entered into the Saudi Acute Myocardial Infarction Registry (STARS). As part of our study, we enrolled 1,471 patients with STEMI. This prospective, multi-center, STARS sub-study included all the consecutive hospital admissions of patients with STEMI. All the relevant clinical data were registered online. The patients were divided into three groups: group 1 received PPCI, group 2 underwent revascularization using thrombolytic therapy (including pharmaco-invasive and rescue or facilitated PPCI), and group 3 received a conservative approach (due to failed thrombolysis or late presentation or contraindications to an intervention). Patients were initially interviewed at the outpatient department in person, and telephonically after one month and one year, as previously described [4].

Categorical data were summarized with absolute frequency and percentages, and continuous data were presented as mean and standard deviation (SD) or median and interquartile range (IQR). A comparison of the three groups, for categorical variables, was done using a chi-square test or Fisher’s exact test; for continuous data, a student's t-test or the Mann-Whitney U test was used. All the analysis was performed using SAS version 9.4 (SAS Institute, Inc, Cary, NC) and R software (R Foundation for Statistical Computing, Vienna, Austria).

## Results

In total, 1,471 patients were included from May 2015 to January 2017. The mean age was 54 ±12 years; 51% were Saudi citizens, and the majority (89%) were male. Their background revealed a high coronary risk profile with 48% diagnosed with diabetes mellitus (DM) and 44% with hypertension (HTN); 54% were active or ex-smokers, and 30% had a high lipid profile. A majority (74%) of the patients were overweight. For almost half of the patients (42%), a PPCI was performed, while 29% received reperfusion by thrombolytic therapy, and a conservative approach was followed in 29%. Patients with a stroke were treated conservatively due to the risk of bleeding. Patients who had a PPCI were mostly hypertensive with recurrent angina and had a history of prior revascularization with PPCI or coronary artery bypass grafting (CABG). The crude all-cause mortality at one year was 11%; it was 7% at one month for group 1, 8% for group 2, and 9% for group 3, which was not statistically significant.

Saudi nationals were more likely to be treated with PPCI, rather than thrombolytic therapy. South Asian nationals and obese patients constituted large proportions of the sample (Table 1). Half of the STEMI was due to an anterior MI, which may explain the subsequent left ventricle (LV) dysfunction. A history of recurrent angina was noted in 20%, and the atypical form of angina or angina equivalent occurred in 6% and 20% respectively. This is noteworthy as a large section of the cohort experienced epigastric or shoulder pain or presented with angina equivalent, in the form of dyspnea. These symptoms may mislead an emergency physician and require a vigilant and thorough assessment.

**Table 1:** Baseline characteristics of the three groups PPCI: primary percutaneous coronary intervention; SD: standard deviation; BMI: body mass index; STEMI: ST-elevation myocardial infarction; CABG: coronary artery bypass grafting; DM: diabetes mellitus; HTN: hypertension; SOB: shortness of breath; OPD: outpatient department; HR: heart rate; SBP: systolic blood pressure; LV: left ventricular; EF: ejection fraction

Variables	PPCI - group 1	Thrombolysis - group 2	Conservative approach - group 3	Total	P-value
Age in years, mean ±SD	55.18 ±12.35	52.11 ±11.66	55.77 ±13.43	54.46 ±12.56	
Males, n (%)	549 (88.41%)	398 (93.21%)	368 (87.00%)	1,315 (89.39%)	0.008
Nationality					
Saudi, n (%)	367 (59.10%)	138 (32.32%)	254 (60.05%)	759 (51.60%)	
Non-Saudi, n (%)	254 (40.90%)	289 (67.68%)	169 (39.95%)	712 (48.40%)	
Ethnicity					
Arab, n (%)	442 (71.18%)	206 (48.24%)	303 (71.63%)	951 (64.65%)	
South Asian (India, Pakistan, Nepal, Bangladesh), n (%)	163 (26.25%)	203 (47.54%)	107 (25.30%)	473 (32.15%)	
Others, n (%)	16 (2.58%)	18 (4.22%)	13 (3.07%)	47 (3.20%)	
BMI, kg/m^2^, mean ±SD	28.10 ±4.82	28.20 ±5.20	28.13 ±5.96	28.14 ±5.28	0.956
Type of STEMI					
Anterior, n (%)	321 (51.69%)	213 (49.88%)	245 (57.92%)	779 (52.96%)	0.03
Inferior, n (%)	232 (37.36%)	181 (42.39%)	140 (33.10%)	553 (37.59%)	
Other, n (%)	68 (10.95%)	33 (7.73%)	38 (8.98%)	139 (9.45%)	
History of angina, n (%)	94 (15.14%)	47 (11.01%)	60 (14.18%)	201 (13.66%)	0.15
History of MI, n (%)	62 (9.98%)	26 (6.09%)	32 (7.57%)	120 (8.16%)	0.067
History of MI/angina, n (%)	119 (19.16%)	57 (13.35%)	69 (16.31%)	245 (16.66%)	0.045
History of PPCI, n (%)	70 (11.27%)	19 (4.45%)	10 (2.36%)	99 (6.73%)	
History of CABG, n (%)	11 (1.77%)	0 (0.00%)	4 (0.95%)	15 (1.02%)	0.019
History of heart failure, n (%)	15 (2.42%)	6 (1.41%)	11 (2.60%)	32 (2.18%)	0.424
History of stroke, n (%)	14 (2.25%)	5 (1.17%)	21 (4.96%)	40 (2.72%)	0.002
History of chronic renal failure, n (%)	21 (3.38%)	9 (2.11%)	18 (4.26%)	48 (3.26%)	0.207
DM, n (%)	305 (49.11%)	181 (42.39%)	225 (53.19%)	711 (48.33%)	0.006
HTN, n (%)	284 (45.73%)	159 (37.24%)	206 (48.70%)	649 (44.12%)	0.002
Hypercholesterolemia, n (%)	197 (31.72%)	130 (30.44%)	110 (26.00%)	437 (29.71%)	0.129
Current/ex-smoking, n (%)	332 (53.46%)	246 (57.61%)	216 (51.06%)	794 (53.98%)	0.151
Chief complaint					
Chest pain, n (%)	576 (92.75%)	404 (94.61%)	368 (87.00%)	1,348 (91.64%)	0.002
SOB/fatigue, n (%)	10 (1.61%)	11 (2.58%)	20 (4.73%)	41 (2.79%)	
Epigastric/shoulder/back/neck pain, n (%)	28 (4.51%)	11 (2.58%)	26 (6.15%)	65 (4.42%)	
Cardiac arrest, n (%)	4 (0.64%)	1 (0.23%)	4 (0.95%)	9 (0.61%)	
Others, n (%)	3 (0.48%)	0 (0.00%)	5 (1.18%)	8 (0.54%)	
First medical contact before presenting to hospital, n (%)	272 (43.80%)	54 (12.65%)	272 (64.30%)	598 (40.65%)	
Visited an emergency department, n (%)	245 (90.07%)	17 (31.48%)	259 (95.22%)	521 (87.12%)	
Clinic doctor (OPD) visit, n (%)	38 (13.97%)	37 (68.52%)	31 (11.40%)	106 (17.73%)	
Visited a pharmacy, n (%)	4 (1.47%)	0 (0.00%)	3 (1.10%)	7 (1.17%)	0.65
Transferred by Red Crescent, n (%)	30 (4.83%)	19 (4.45%)	25 (5.91%)	74 (5.03%)	0.595
HR (bpm) upon arrival, mean ±SD	85.62 ±25.86	84.99 ±18.20	85.57 ±19.36	85.42 ±22.04	0.889
SBP (mmHg) upon arrival, mean ±SD	132.0 ±28.65	132.6 ±31.17	127.0 ±24.59	130.7 ±28.41	0.005
HR of >100 bpm, n (%)	108 (17.39%)	67 (15.69%)	73 (17.26%)	248 (16.86%)	0.745
SBP of <90 mmHg, n (%)	26 (4.19%)	19 (4.45%)	12 (2.84%)	57 (3.87%)	0.414
Cardiac arrest upon arrival, n (%)	18 (2.90%)	18 (4.22%)	14 (3.31%)	50 (3.40%)	0.509
Killip class					
Class I, n (%)	563 (90.66%)	349 (81.73%)	348 (82.27%)	1,260 (85.66%)	
Class II/III, n (%)	35 (5.64%)	69 (16.16%)	64 (15.13%)	168 (11.42%)	
Class IV. n (%)	23 (3.70%)	9 (2.11%)	11 (2.60%)	43 (2.92%)	
Echo report					
Normal LV systolic function (EF of >50%), n (%)	126 (24.71%)	108 (27.76%)	92 (22.94%)	326 (25.08%)	0.012
Mild LV systolic dysfunction (EF of 40-50%), n (%)	168 (32.94%)	155 (39.85%)	144 (35.91%)	467 (35.92%)	
Moderate LV systolic dysfunction (EF of 30-40%), n (%)	148 (29.02%)	101 (25.96%)	115 (28.68%)	364 (28.00%)	
Severe LV systolic dysfunction (EF of <30%), n (%)	68 (13.33%)	25 (6.43%)	50 (12.47%)	143 (11.00%)	

In the cohort, a small proportion (3%) had a history of stroke or renal disease. As in other studies conducted in Saudi Arabia, half of these patients were diabetic. A concern was that the proportion of smokers (53%) was higher than previously documented (Table 2). A small proportion (5%) of the STEMI patients presented with cardiogenic shock and 5% had pulmonary edema. Also concerning was that only one-fifth had a preserved LV systolic function on admission.

**Table 2:** MACE in the three groups at one month MACE: major adverse cardiovascular events; PPCI: primary percutaneous coronary intervention; MI: myocardial infarction; VT: ventricular tachycardia; VF: ventricular fibrillation

Variables	PPCI - group 1, n (%)	Thrombolysis - group 2, n (%)	Conservative approach - group 3, n (%)	Total, n (%)	P-value
Recurrent ischemia	13 (2.09%)	55 (12.88%)	30 (7.09%)	98 (6.66%)	
Recurrent MI	7 (1.13%)	12 (2.81%)	12 (2.84%)	31 (2.11%)	0.082
Atrial fibrillation/flutter	13 (2.09%)	16 (3.75%)	11 (2.60%)	40 (2.72%)	0.266
Heart failure	56 (9.02%)	47 (11.01%)	53 (12.53%)	156 (10.61%)	0.185
Cardiogenic shock	52 (8.37%)	24 (5.62%)	32 (7.57%)	108 (7.34%)	0.239
VT/VF arrest	35 (5.64%)	27 (6.32%)	22 (5.20%)	84 (5.71%)	0.776
Stroke	4 (0.64%)	2 (0.47%)	4 (0.95%)	10 (0.68%)	0.692
Major bleeding	10 (1.61%)	3 (0.70%)	6 (1.42%)	19 (1.29%)	0.425
Stent thrombosis	3 (0.57%)	1 (0.53%)	1 (0.30%)	5 (0.48%)	0.849
Death	20 (3.22%)	16 (3.75%)	24 (5.67%)	60 (4.08%)	0.133

Due to the guideline-directed medical therapy (GDMT), the majority were prescribed beta-blockers and statins, with half of the sample using insulin for DM. Clopidogrel was used more than ticagrelor, as recommended in the 2018 guidelines.

The PPCI strategy significantly reduced recurrent ischemic events compared to the other strategies (p: <0.001). The crude mortality at one year in the PPCI group was 11%; it was 12% for the thrombolysis group and 15% for the conservative approach group. The differences were not statistically significant.

## Discussion

The current study was a sub-study of the STARS, exploring the management strategies used in the STEMI population. The STARS is the latest registry related to healthcare in Saudi Arabia, and it is used by healthcare sectors in all geographic regions. The snap-shot-design used in the STARS allows for obtaining high-quality data, and reduces issues such as missing data and “registry exhaustion”. Regarding the STEMI population in Saudi Arabia, the current study indicated a high prevalence of coronary artery disease risk factors in a younger age group, compared to other registries. It also highlighted a high prevalence of DM, HTN, and smoking, and controlling these risks is crucial for the prevention of acute coronary syndromes [5].

The sub-study demonstrated a notable improvement in the rate of PPCI (42.5%). However, this percentage remains low when compared to countries in Europe and North America. It also indicated that 40% of all the PPCI procedures failed to achieve a door-to-balloon time of less than 90 minutes. The rate of recurrent ischemia was significantly lower in the PPCI group compared to the thrombolysis group. This is attributed to the higher number of catheter laboratories and interventional cardiologists in the country. The lack of any difference in the mortality is possibly related to the late presentation prevalence of acute MI, a higher risk profile, and prior myocardial damage during previous PPCI or CABG. The STEMI network may decrease the deficit [6,7].

PPCI reduces the risk of death and other major adverse cardiac events (MACE) in patients with STEMI. All patients, irrespective of the mode of therapy, received appropriate GDMT at admission and discharge [8,9]. There was a significant difference in the rate of PPCI between Saudis and non-Saudis. This disparity is possibly related to the blue-collar worker health coverage and lack of access to timely care. The language difference may also contribute to the difference, as most of the blue-collar workers are of South Asian origin and do not speak Arabic. The lack of governmental coverage for healthcare to non-Saudis may affect the decision regarding self-presentation due to financial reasons. A clustering model would support such an investigation [10,11].

For the PPCI group, the target time from door-to-needle was 45 minutes (the required ideal maximum time is 30 minutes), and the door-to-balloon time was 55 minutes (the maximum accepted ideal time is 90 minutes). A quality program to improve the cause of the delay is important and should be assessed continuously. Adhering to the ideal periods will decrease the future development of heart failure and decrease morbidity and mortality [12,13]. The in-hospital total mortality rate for STEMI in our series was 8.6% after one month, and 12.2% after one year. Similar findings were reported from the Kerala ACS Registry, 8.2% at one month, but it was higher than the GRACE (7%), the Euro Heart Survey ACS II (6%), and the CREATE registry (8.6%), which included mortality over 30 days [14].

Primary prevention and risk factor control measures had a significant impact on the systolic function. An echocardiographic assessment was done on admission, which may indicate a stunning or hibernating element that could affect the function. The total ischemic time (time between symptom onset and reperfusion therapy) is the most important factor to achieve the best possible outcome for patients with acute MI [12,15]. The LV systolic function at presentation indicated that 25% had a normal systolic function and an ejection fraction (EF) of more than 50%, 35% had mildly reduced LV function (EF of 40-50%), 28% had moderate LV systolic function (EF of 30-40%), and 11% had severe LV systolic dysfunction (EF of less than 30%).

There are several limitations to our study. Firstly, this was a sub-study of the STARS. All the limitations associated with the main paper will apply to the current report as well. Secondly, including only STEMI patients was not pre-specified in the original paper, relegating this report to the status of a post-hoc analysis, useful for theory generation.

## Conclusions

In conclusion, PPCI strategies are an effective method for reducing recurrent ischemic attacks. Access to catheter laboratories should be increased to enhance the role of PPCI in reducing short-term mortality compared to other strategies in the management of STEMI patients in Saudi Arabia.
